# Supplementary material for: Prenatal polycyclic aromatic hydrocarbon exposure and asthma at age 8–9 years in a multi-site longitudinal study
Source: Environ Health. 2024 Mar 8;23:26. doi: 10.1186/s12940-024-01066-2 (PMC10921622; doi:10.1186/s12940-024-01066-2)
Supplement: Supplementary file 1 — Supplementary Material 1. [file 12940_2024_1066_MOESM1_ESM.docx]

Table S1. Descriptive statistics for SG-adjusted OH-PAH concentrations (ng/mL).

| **Population** | **OH-PAH** | **Geometric Mean** | **Geometric SD** | **LOD** | **P25** | **Median** | **P75** | **Max** |
| --- | --- | --- | --- | --- | --- | --- | --- | --- |
| **Overall** | 1-NAP | 0.62 | 4.85 | N/A | 0.31 | 0.67 | 1.55 | 217.0 |
|  | 2-NAP | 3.69 | 2.66 | N/A | 2.17 | 3.85 | 6.63 | 136.8 |
|  | 2-PHEN | 0.08 | 2.22 | N/A | 0.05 | 0.08 | 0.12 | 101.0 |
|  | 3-PHEN | 0.08 | 2.21 | N/A | 0.05 | 0.08 | 0.12 | 56.0 |
|  | 1/9-PHEN | 0.18 | 3.62 | N/A | 0.09 | 0.23 | 0.46 | 11.8 |
| **CANDLE** | 1-NAP | 1.18 | 3.16 | 0.02 | 0.54 | 1.01 | 2.07 | 216.9 |
|  | 2-NAP | 4.82 | 2.35 | 0.025 | 2.81 | 4.83 | 7.74 | 136.8 |
|  | 2-PHEN | 0.09 | 1.92 | 0.03 | 0.06 | 0.09 | 0.13 | 3.7 |
|  | 3-PHEN | 0.10 | 1.90 | 0.03 | 0.06 | 0.09 | 0.14 | 2.7 |
|  | 1/9-PHEN | 0.28 | 3.20 | 0.03 | 0.18 | 0.35 | 0.57 | 11.8 |
| **TIDES** | 1-NAP | 0.19 | 4.90 | 0.04 | 0.06 | 0.24 | 0.56 | 102.6 |
|  | 2-NAP | 2.28 | 2.72 | 0.017 | 1.19 | 2.32 | 4.27 | 29.9 |
|  | 2-PHEN | 0.06 | 2.50 | 0.003 | 0.03 | 0.05 | 0.09 | 101.0 |
|  | 3-PHEN | 0.05 | 2.40 | 0.003 | 0.03 | 0.05 | 0.08 | 56.0 |
|  | 1/9-PHEN | 0.09 | 3.13 | 0.007 | 0.04 | 0.10 | 0.18 | 1.7 |

Note: Values below the LOD were imputed with LOD/√2 for descriptive statistics

Table S2. Detection rates of OH-PAHs in the study population. PAH metabolites with detection rates >60% (blue highlight) in both cohorts were included in the analysis.

| **OH-PAH** | **Overall**  **(N=1,081)** | **CANDLE**  **(N=698)** | **TIDES**  **(N=383)** | **TIDES – UCSF**  **(N=79)** | **TIDES – UMN**  **(N=118)** | **TIDES – URMC**  **(N=98)** | **TIDES – UW**  **(N=93)** |
| --- | --- | --- | --- | --- | --- | --- | --- |
| **1-NAP** | 0.88 | 1.00 | 0.66 | 0.80 | 0.59 | 0.56 | 0.73 |
| **2-NAP** | 1.00 | 1.00 | 0.99 | 1.00 | 0.99 | 0.99 | 1.00 |
| **2-PHEN** | 0.91 | 0.87 | 0.98 | 1.00 | 0.97 | 0.99 | 0.96 |
| **3-PHEN** | 0.90 | 0.87 | 0.97 | 1.00 | 0.95 | 0.99 | 0.95 |
| **4-PHEN** | 0.50 | 0.42 | 0.64 | 0.72 | 0.55 | 0.80 | 0.54 |
| **1/9-PHEN** | 0.85 | 0.84 | 0.88 | 0.94 | 0.80 | 0.92 | 0.90 |
| **2/3/9-FLUO** | 0.73 | 0.97 | 0.30 | 0.34 | 0.20 | 0.45 | 0.23 |
| **1-PYR** | 0.73 | 0.89 | 0.43 | 0.46 | 0.36 | 0.66 | 0.27 |
| **1-BAA** | 0.00 | 0.00 | 0.01 | 0.01 | 0.02 | 0.00 | 0.00 |
| **3-BCP** | 0.00 | 0.00 | 0.00 | 0.00 | 0.00 | 0.00 | 0.00 |
| **1-CHRY** | 0.01 | 0.00 | 0.02 | 0.03 | 0.00 | 0.01 | 0.03 |
| **6-CHRY** | 0.01 | 0.00 | 0.02 | 0.08 | 0.01 | 0.01 | 0.00 |

| Table S3. Characteristics of CANDLE and TIDES participants included and excluded from the study population   \|  \| Excluded (N=1322) \| Included (N=1081) \| \| --- \| --- \| --- \| \| **Cohort** \|  \|  \| \| CANDLE \| 805 (60.9%) \| 698 (64.6%) \| \| TIDES \| 517 (39.1%) \| 383 (35.4%) \| \| **Child sex** \|  \|  \| \| F \| 575 (43.5%) \| 559 (51.7%) \| \| M \| 610 (46.1%) \| 522 (48.3%) \| \| Missing \| 137 (10.4%) \| 0 (0%) \| \| **Child age at clinic visit** \|  \|  \| \| 8 \| 126 (9.5%) \| 605 (56.0%) \| \| 9 \| 91 (6.9%) \| 376 (34.8%) \| \| 10 \| 50 (3.8%) \| 100 (9.3%) \| \| 11 \| 2 (0.2%) \| 0 (0%) \| \| Missing \| 1053 (79.7%) \| 0 (0%) \| \| **Postnatal second-hand smoke exposure** \|  \|  \| \| Yes \| 203 (15.4%) \| 170 (15.7%) \| \| No \| 451 (34.1%) \| 865 (80.0%) \| \| Missing \| 668 (50.5%) \| 46 (4.3%) \| \| **First born** \|  \|  \| \| Yes \| 591 (44.7%) \| 476 (44.0%) \| \| No \| 645 (48.8%) \| 596 (55.1%) \| \| Missing \| 86 (6.5%) \| 9 (0.8%) \| \| **Maternal race** \|  \|  \| \| Black or African American \| 585 (44.3%) \| 471 (43.6%) \| \| Multiple races \| 67 (5.1%) \| 47 (4.3%) \| \| White \| 560 (42.4%) \| 504 (46.6%) \| \| Other \| 83 (6.3%) \| 55 (5.1%) \| \| Missing \| 27 (2.0%) \| 4 (0.4%) \| \| **Maternal ethnicity** \|  \|  \| \| Not Hispanic or Latino \| 1226 (92.7%) \| 1042 (96.4%) \| \| Hispanic or Latino \| 79 (6.0%) \| 35 (3.2%) \| \| Missing \| 17 (1.3%) \| 4 (0.4%) \| \| **Maternal education at enrollment** \|  \|  \| \| Less than high school \| 179 (13.5%) \| 76 (7.0%) \| \| High school completion \| 523 (39.6%) \| 355 (32.8%) \| \| Graduated college or technical school \| 348 (26.3%) \| 365 (33.8%) \| \| Some or more graduate school \| 257 (19.4%) \| 281 (26.0%) \| \| Missing \| 15 (1.1%) \| 4 (0.4%) \| \| **Maternal age at enrollment** \|  \|  \| \| Mean (SD) \| 27.2 (5.96) \| 28.3 (5.88) \| \| Median [Min, Max] \| 27.0 [16.0, 45.0] \| 29.0 [16.0, 44.0] \| \| Missing \| 29 (2.2%) \| 1 (0.1%) \| \| **Maternal history of asthma** \|  \|  \| \| Yes \| 115 (8.7%) \| 181 (16.7%) \| \| No \| 531 (40.2%) \| 868 (80.3%) \| \| Missing \| 676 (51.1%) \| 32 (3.0%) \| \| **Maternal urinary cotinine (ng/mL)** \|  \|  \| \| Median [Min, Max] \| 0.381 [0.00141, 5010] \| 0.0937 [0.00141, 197] \| \| Missing \| 546 (41.3%) \| 4 (0.4%) \| \| **RPP-adjusted household income (USD)** \|  \|  \| \| Mean (SD) \| 52500 (49100) \| 66000 (54700) \| \| Median [Min, Max] \| 37400 [2490, 213000] \| 51600 [2490, 213000] \| \| Missing \| 725 (54.8%) \| 62 (5.7%) \|   Table S4. Correlation coefficients between OH-PAH concentrations.   \| **OH-PAH** \| **1-NAP** \| **2-NAP** \| **2-PHEN** \| **3-PHEN** \| **1/9-PHEN** \| \| --- \| --- \| --- \| --- \| --- \| --- \| \| **1-NAP** \| 1 \| 0.40 \| 0.34 \| 0.43 \| 0.45 \| \| **2-NAP** \|  \| 1 \| 0.36 \| 0.43 \| 0.36 \| \| **2-PHEN** \|  \|  \| 1 \| 0.74 \| 0.55 \| \| **3-PHEN** \|  \|  \|  \| 1 \| 0.58 \| \| **1/9-PHEN** \|  \|  \|  \|  \| 1 \|   Note: Values below the LOD were imputed with LOD/√2 for descriptive statistics  Table S5. Characteristics of the study population by tertile of total OH-PAH exposure (sum of SG-adjusted OH-PAH metabolites). | | | | |
| --- | --- | --- | --- | --- | --- | --- | --- | --- | --- | --- | --- | --- | --- | --- | --- | --- | --- | --- | --- | --- | --- | --- | --- | --- | --- | --- | --- | --- | --- | --- | --- | --- | --- | --- | --- | --- | --- | --- | --- | --- | --- | --- | --- | --- | --- | --- | --- | --- | --- | --- | --- | --- | --- | --- | --- | --- | --- | --- | --- | --- | --- | --- | --- | --- | --- | --- | --- | --- | --- | --- | --- | --- | --- | --- | --- | --- | --- | --- | --- | --- | --- | --- | --- | --- | --- | --- | --- | --- | --- | --- | --- | --- | --- | --- | --- | --- | --- | --- | --- | --- | --- | --- | --- | --- | --- | --- | --- | --- | --- | --- | --- | --- | --- | --- | --- | --- | --- | --- | --- | --- | --- | --- | --- | --- | --- | --- | --- | --- | --- | --- | --- | --- | --- | --- | --- | --- | --- | --- | --- | --- | --- | --- | --- | --- | --- | --- | --- | --- | --- | --- | --- | --- | --- | --- | --- | --- | --- | --- | --- | --- | --- | --- | --- | --- | --- | --- | --- | --- | --- | --- | --- | --- | --- | --- | --- | --- | --- | --- | --- | --- | --- | --- | --- | --- | --- | --- | --- | --- | --- | --- | --- | --- | --- | --- | --- | --- | --- | --- | --- |
|  | PAH exposure tertile | | |  |
|  | Tertile 1 (N=361) | Tertile 2 (N=361) | Tertile 3 (N=359) | Overall (N=1081) |
| **Cohort** |  |  |  |  |
| CANDLE | 138 (38.2%) | 271 (75.3%) | 289 (80.3%) | 698 (64.6%) |
| TIDES | 223 (61.8%) | 89 (24.7%) | 71 (19.7%) | 383 (35.4%) |
| **Child sex** |  |  |  |  |
| F | 191 (52.9%) | 196 (54.4%) | 172 (47.8%) | 559 (51.7%) |
| M | 170 (47.1%) | 164 (45.6%) | 188 (52.2%) | 522 (48.3%) |
| **Child age at clinic visit** |  |  |  |  |
| 8 | 191 (52.9%) | 195 (54.2%) | 219 (60.8%) | 604 (56.0%) |
| 9 | 143 (39.6%) | 132 (36.7%) | 101 (28.1%) | 376 (34.8%) |
| 10 | 27 (7.5%) | 33 (9.2%) | 40 (11.1%) | 100 (9.3%) |
| **Postnatal second-hand smoke exposure** |  |  |  |  |
| Yes | 23 (6.4%) | 73 (20.3%) | 74 (20.6%) | 170 (15.7%) |
| No | 320 (88.6%) | 269 (74.7%) | 276 (76.7%) | 865 (80.0%) |
| Missing | 18 (5.0%) | 18 (5.0%) | 10 (2.8%) | 46 (4.3%) |
| **First born** |  |  |  |  |
| Yes | 170 (47.1%) | 152 (42.2%) | 154 (42.8%) | 476 (44.0%) |
| No | 187 (51.8%) | 205 (56.9%) | 204 (56.7%) | 596 (55.1%) |
| Missing | 4 (1.1%) | 3 (0.8%) | 2 (0.6%) | 9 (0.8%) |
| **Maternal race** |  |  |  |  |
| Black or African American | 66 (18.3%) | 178 (49.4%) | 227 (63.1%) | 471 (43.6%) |
| Multiple races | 14 (3.9%) | 16 (4.4%) | 17 (4.7%) | 47 (4.3%) |
| White | 251 (69.5%) | 150 (41.7%) | 103 (28.6%) | 504 (46.6%) |
| Other | 28 (7.8%) | 16 (4.4%) | 11 (3.1%) | 55 (5.1%) |
| Missing | 2 (0.6%) | 0 (0%) | 2 (0.6%) | 4 (0.4%) |
| **Maternal ethnicity** |  |  |  |  |
| Not Hispanic or Latino | 347 (96.1%) | 348 (96.7%) | 347 (96.4%) | 1042 (96.4%) |
| Hispanic or Latino | 13 (3.6%) | 12 (3.3%) | 10 (2.8%) | 35 (3.2%) |
| Missing | 1 (0.3%) | 0 (0%) | 3 (0.8%) | 4 (0.4%) |
| **Maternal education at enrollment** |  |  |  |  |
| Less than high school | 14 (3.9%) | 24 (6.7%) | 38 (10.6%) | 76 (7.0%) |
| High school completion | 50 (13.9%) | 150 (41.7%) | 155 (43.1%) | 355 (32.8%) |
| Graduated college or technical school | 132 (36.6%) | 118 (32.8%) | 115 (31.9%) | 365 (33.8%) |
| Some or more graduate school | 163 (45.2%) | 68 (18.9%) | 50 (13.9%) | 281 (26.0%) |
| Missing | 2 (0.6%) | 0 (0%) | 2 (0.6%) | 4 (0.4%) |
| **Maternal age at enrollment** |  |  |  |  |
| Mean (SD) | 30.3 (5.55) | 27.5 (5.90) | 27.1 (5.65) | 28.3 (5.88) |
| Median [Min, Max] | 31.0 [16.0, 43.0] | 28.0 [16.0, 44.0] | 27.0 [16.0, 42.0] | 29.0 [16.0, 44.0] |
| Missing | 1 (0.3%) | 0 (0%) | 0 (0%) | 1 (0.1%) |
| **Maternal history of asthma** |  |  |  |  |
| Yes | 56 (15.5%) | 58 (16.1%) | 67 (18.6%) | 181 (16.7%) |
| No | 299 (82.8%) | 287 (79.7%) | 282 (78.3%) | 868 (80.3%) |
| Missing | 6 (1.7%) | 15 (4.2%) | 11 (3.1%) | 32 (3.0%) |
| **Maternal urinary cotinine (ng/mL)** |  |  |  |  |
| Median [Min, Max] | 0.00919 [0.00141, 159] | 0.163  [0.00141, 75.6] | 0.220  [0.00141, 197] | 0.0937 [0.00141, 197] |
| Missing | 1 (0.3%) | 2 (0.6%) | 1 (0.3%) | 4 (0.4%) |
| **RPP-adjusted household income (USD)** |  |  |  |  |
| Mean (SD) | 92600 (57700) | 56000 (50500) | 48700 (44400) | 66000 (54700) |
| Median [Min, Max] | 81400  [2490, 213000] | 40700  [2550, 213000] | 31000  [2490, 199000] | 51600  [2490, 213000] |
| Missing | 15 (4.2%) | 20 (5.6%) | 27 (7.5%) | 62 (5.7%) |

Table S6. Metabolite weights contributing to the WQS Index for minimally- and fully-adjusted WQS models, where associations are constrained to be positive or negative.

|  | **Positive** | | **Negative** | |
| --- | --- | --- | --- | --- |
| **OH-PAH** | **Minimal** | **Full** | **Minimal** | **Full** |
| 1-NAP | 0.008 | 0.001 | 0.80 | 0.57 |
| 2-NAP | 0.36 | 0.06 | 0.03 | 0.15 |
| 2-PHEN | 0.19 | 0.37 | 0.03 | 0.003 |
| 3-PHEN | 0.05 | 0.004 | 0.13 | 0.27 |
| 1/9-PHEN | 0.39 | 0.56 | 0.02 | 0.008 |

Table S7. Associations between **mutually-adjusted** prenatal OH-PAH metabolites and asthma at age 8-9, wheezing trajectories in childhood, and asthma phenotypes at age 8-9. Adjusted odds ratios (OR) and 95 % confidence intervals (CI) associated with a twofold increase in individual OH-PAH metabolite were determined by logistic regression (asthma outcomes) and multinomial regression (wheezing trajectories).

|  |  | Mutually-adjusted metabolite model | | Individual metabolite models (primary analysis) | |
| --- | --- | --- | --- | --- | --- |
| **Outcome** | **OH-PAH** | **OR** | **95% CI** | **OR** | **95% CI** |
| **Asthma (8-9y)** | 1-NAP | **0.83** | **(0.71, 0.96)** | **0.86** | **(0.76, 0.98)** |
|  | 2-NAP | 1.03 | (0.87, 1.21) | 0.96 | (0.83, 1.12) |
|  | 2-PHEN | 1.04 | (0.85, 1.28) | 0.99 | (0.82, 1.20) |
|  | 3-PHEN | 0.81 | (0.58, 1.12) | 0.90 | (0.71, 1.15) |
|  | 1/9-PHEN | **1.20** | **(1.01, 1.44)** | 1.07 | (0.95, 1.21) |
| **Wheezing trajectories** | | | |  |  |
| Early wheezing | 1-NAP | 1.01 | (0.91, 1.12) | 0.98 | (0.89, 1.08) |
|  | 2-NAP | 0.97 | (0.84, 1.12) | 0.93 | (0.81, 1.07) |
|  | 2-PHEN | 1.00 | (0.79, 1.28) | 0.90 | (0.75, 1.07) |
|  | 3-PHEN | 0.86 | (0.65, 1.14) | 0.85 | (0.70, 1.03) |
|  | 1/9-PHEN | 1.03 | (0.92, 1.16) | 0.98 | (0.88, 1.08) |
| Late wheezing | 1-NAP | 0.98 | (0.85, 1.12) | 0.98 | (0.86, 1.11) |
|  | 2-NAP | 1.09 | (0.90, 1.32) | 1.03 | (0.87, 1.23) |
|  | 2-PHEN | 0.99 | (0.69, 1.42) | 0.92 | (0.73, 1.16) |
|  | 3-PHEN | 0.83 | (0.56, 1.21) | 0.88 | (0.69, 1.12) |
|  | 1/9-PHEN | 1.02 | (0.87, 1.20) | 0.98 | (0.86, 1.13) |
| Persistent wheezing | 1-NAP | 0.89 | (0.75, 1.05) | 0.92 | (0.79, 1.07) |
|  | 2-NAP | 1.09 | (0.86, 1.38) | 0.99 | (0.80, 1.23) |
|  | 2-PHEN | 1.09 | (0.79, 1.51) | 0.97 | (0.74, 1.28) |
|  | 3-PHEN | 0.66 | (0.42, 1.01) | 0.88 | (0.65, 1.19) |
|  | 1/9-PHEN | **1.32** | **(1.04, 1.67)** | 1.12 | (0.95, 1.33) |
| **Secondary outcomes** | |  |  |  |  |
| Asthma with recent exacerbation | 1-NAP | 0.82 | (0.65, 1.05) | **0.76** | **(0.61, 0.96)** |
|  | 2-NAP | 0.92 | (0.74, 1.15) | 0.81 | (0.66, 1.01) |
|  | 2-PHEN | 0.89 | (0.54, 1.48) | 0.77 | (0.52, 1.16) |
|  | 3-PHEN | 0.80 | (0.46, 1.39) | 0.73 | (0.52, 1.03) |
|  | 1/9-PHEN | 1.21 | (0.97, 1.51) | 1.02 | (0.87, 1.21) |
| Asthma with other atopic diseases | 1-NAP | **0.79** | **(0.65, 0.95)** | **0.80** | **(0.69, 0.94)** |
|  | 2-NAP | 0.96 | (0.79, 1.15) | 0.89 | (0.75, 1.05) |
|  | 2-PHEN | 1.00 | (0.80, 1.24) | 0.93 | (0.73, 1.18) |
|  | 3-PHEN | 0.85 | (0.60, 1.21) | 0.85 | (0.65, 1.11) |
|  | 1/9-PHEN | 1.19 | (0.98, 1.46) | 1.04 | (0.91, 1.18) |

Table S8. Cohort-specific associations between prenatal OH-PAH metabolites and asthma at age 8-9 in childhood. Adjusted odds ratios (OR) and 95 % confidence intervals (CI) associated with a twofold increase in individual OH-PAH metabolite were determined by fully-adjusted logistic regression models.w

|  | **CANDLE** | | **TIDES** | |
| --- | --- | --- | --- | --- |
| **OH-PAH** | **OR** | **95% CI** | **OR** | **95% CI** |
| 1-NAP | **0.83** | **(0.71, 0.96)** | 0.83 | (0.64, 1.08) |
| 2-NAP | 0.83 | (0.66, 1.03) | 1.29 | (0.88, 1.88) |
| 2-PHEN | 0.86 | (0.64, 1.17) | 1.16 | (0.85, 1.58) |
| 3-PHEN | **0.71** | **(0.51, 0.99)** | 1.48 | (0.83, 2.67) |
| 4-PHEN | - | - | **1.75** | **(1.07, 2.86)** |
| 1/9-PHEN | 1.01 | (0.88, 1.15) | **1.49** | **(1.05, 2.12)** |
| 2/3/9-FLUO | 0.85 | (0.67, 1.08) | - | - |
| 1-PYR | 0.95 | (0.70, 1.27) | - | - |

Table S9. Associations between prenatal OH-PAH metabolites and asthma at age 8-9 in childhood after exclusion of individual TIDES study sites in fully-adjusted logistic regression models.

|  | **Excluding  San Francisco, CA** | | **Excluding  Minneapolis, MN** | | **Excluding  Seattle, WA** | | **Excluding Rochester, NY** | |
| --- | --- | --- | --- | --- | --- | --- | --- | --- |
| **OH-PAH** | **OR** | **95% CI** | **OR** | **95% CI** | **OR** | **95% CI** | **OR** | **95% CI** |
| 1-NAP | 0.88 | (0.78, 1.00) | **0.83** | **(0.72, 0.96)** | **0.85** | **(0.75, 0.97)** | **0.85** | **(0.74, 0.97)** |
| 2-NAP | 0.95 | (0.81, 1.1) | 0.93 | (0.79, 1.09) | 0.96 | (0.82, 1.12) | 0.90 | (0.77, 1.07) |
| 2-PHEN | 0.99 | (0.82, 1.21) | 0.92 | (0.72, 1.18) | 0.99 | (0.81, 1.20) | 0.96 | (0.78, 1.18) |
| 3-PHEN | 0.91 | (0.72, 1.16) | 0.78 | (0.59, 1.04) | 0.90 | (0.70, 1.15) | 0.86 | (0.67, 1.11) |
| 1/9-PHEN | 1.08 | (0.96, 1.23) | 1.02 | (0.90, 1.16) | 1.06 | (0.94, 1.20) | 1.05 | (0.93, 1.19) |

Table S10. Associations between prenatal OH-PAH metabolites and asthma at age 8-9 in childhood in primary and sensitivity analyses in fully-adjusted logistic regression models.

| **Analysis** | **PAH metabolite** | **OR** | **95% CI** |
| --- | --- | --- | --- |
| Primary analysis | 1-NAP | **0.86** | **(0.76, 0.98)** |
|  | 2-NAP | 0.96 | (0.83, 1.12) |
|  | 2-PHEN | 0.99 | (0.82, 1.20) |
|  | 3-PHEN | 0.90 | (0.71, 1.15) |
|  | 1/9-PHEN | 1.07 | (0.95, 1.21) |
| Imputation of OH-PAH concentrations below the detection limit with censored maximum likelihood imputation (CLMI) | 1-NAP | 0.92 | (0.81, 1.04) |
|  | 2-NAP | 0.96 | (0.80, 1.14) |
|  | 2-PHEN | 1.00 | (0.80, 1.25) |
|  | 3-PHEN | 0.90 | (0.70, 1.14) |
|  | 1/9-PHEN | 1.07 | (0.94, 1.22) |
| SG-adjusted OH-PAH metabolites | 1-NAP | **0.87** | **(0.76, 0.98)** |
|  | 2-NAP | 0.97 | (0.83, 1.13) |
|  | 2-PHEN | 1.01 | (0.83, 1.22) |
|  | 3-PHEN | 0.92 | (0.73, 1.17) |
|  | 1/9-PHEN | 1.08 | (0.95, 1.22) |
| Additional adjustment by birth weight, gestational age, and birth year | 1-NAP | **0.86** | **(0.76, 0.99)** |
|  | 2-NAP | 0.95 | (0.82, 1.11) |
|  | 2-PHEN | 0.99 | (0.81, 1.22) |
|  | 3-PHEN | 0.89 | (0.69, 1.14) |
|  | 1/9-PHEN | 1.08 | (0.95, 1.22) |
| Imputation of missing covariates with multiple imputation by chained equations (MICE) | 1-NAP | **0.87** | **(0.78, 0.98)** |
|  | 2-NAP | 0.98 | (0.86, 1.12) |
|  | 2-PHEN | 0.95 | (0.79, 1.15) |
|  | 3-PHEN | 0.90 | (0.73, 1.10) |
|  | 1/9-PHEN | 1.06 | (0.95, 1.19) |
| Inverse probability of selection weighting | 1-NAP | **0.86** | **(0.75, 0.98)** |
|  | 2-NAP | 0.98 | (0.84, 1.14) |
|  | 2-PHEN | 0.99 | (0.82, 1.20) |
|  | 3-PHEN | 0.92 | (0.72, 1.17) |
|  | 1/9-PHEN | 1.07 | (0.94, 1.21) |
